# Frequent, quantitative bone planar scintigraphy for determination of bone anabolism in growing mice

**DOI:** 10.7717/peerj.12355

**Published:** 2021-12-09

**Authors:** Ariane Zaloszyc, Claus Peter Schmitt, Amira Sayeh, Laetitia Higel, Catherine-Isabelle Gros, Fabien Bornert, Gaëlle Aubertin-Kirch, Jean-Philippe Dillenseger, Christian Goetz, André Constantinesco, Michel Fischbach, Seiamak Bahram, Philippe Choquet

**Affiliations:** 1 Service de Pédiatrie 1, Hôpital de Hautepierre, Hôpitaux Universitaires de Strasbourg, Strasbourg, France; 2 Fédération Hospitalo-Universitaire, OMICARE, Centre de Recherche d’Immunologie et d’Hématologie, Strasbourg, France; 3 INSERM UMR_S 1109, Immuno Rhumatologie Moléculaire, Centre de Recherche d’Immunologie et d’Hématologie, Strasbourg, France; 4 Imagerie Préclinique—UF6237, Pôle d’imagerie, Hôpitaux Universitaires de Strasbourg, Strasbourg, France; 5 Center for Pediatric and Adolescent Medicine, Division of Pediatric Nephrology, University of Heidelberg, Heidelberg, Germany; 6 Pôle de Médecine et Chirurgie Bucco-dentaires, Hôpitaux Universitaires de Strasbourg, Strasbourg, France; 7 Faculté de chirurgie dentaire, Université de Strasbourg, Strasbourg, France; 8 INSERM UMR 1260, Regenerative Nanomedicine (RNM), FMTS, Université de Strasbourg, Strasbourg, France; 9 Medical Image Analysis center (MIAC AG), Basel, Switzeland; 10 ICube, UMR 7357 CNRS, Université de Strasbourg, Strasbourg, France; 11 Department of Nuclear Medicine, Medical Center–University of Freiburg, Klinik für Nuklear Medizin, Freiburg, Germany; 12 Plateforme GENOMAX, Laboratoire d’Immuno Rhumatologie Moléculaire, INSERM UMR_S1109, LabEx Transplantex, Centre de Recherche d’Immunologie et d’Hématologie, Faculté de Médecine, FMTS, Université de Strasbourg, Strasbourg, France; 13 Franco-Japanese Nextgen HLA Laboratory, INSERM, Strasbourg and Nagano, France, Japan; 14 Laboratoire Central d’Immunologie, Plateau Technique de Biologie, Pôle de Biologie, Nouvel Hôpital Civil, Strasbourg, Strasbourg, France

**Keywords:** Radionuclide imaging, Mice, Subcutaneous application, Growth

## Abstract

**Background:**

To provide insight into bone turnover, quantitative measurements of bone remodeling are required. Radionuclide studies are widely used in clinical care, but have been rarely used in the exploration of the bone in preclinical studies. We describe a bone planar scintigraphy method for frequent assessment of bone activity in mice across the growing period. Since repeated venous radiotracer injections are hardly feasible in mice, we investigated the subcutaneous route.

**Methods:**

Repeated ^99m^Tc-hydroxymethylene diphosphonate (HMDP) tracer bone planar scintigraphy studies of the knee region and µCT to measure femur growth rate were performed in eight mice between week 6 and week 27 of life, *i.e.*, during their growth period. Three independent investigators assessed the regions of interest (ROI). An index was calculated based on the counts in knees ROI (normalized by pixels and seconds), corrected for the activity administered, the decay between administration and imaging, and individual weights.

**Results:**

A total of 93 scintigraphy studies and 85 µCT were performed. Repeated subcutaneous tracer injections were well tolerated and allowed for adequate radionuclide studies. Mean scintigraphic indexes in the knees ROI decreased from 87.4 ± 2.6 × 10^−6^ counts s^−1^ pixel^−1^ MBq^−1^ g^−1^ at week 6 to 15.0 ± 3.3 × 10^−6^ counts s^−1^ pixel^−1^ MBq^−1^ g^−1^ at week 27. The time constant of the fitted exponential decay was equal to 23.5 days. As control mean femur length assessed by µCT increased from 12.2 ± 0.8 mm at week 6 to 15.8 ± 0.2 mm at week 22. The time constant of the fitted Gompertz law was equal to 26.7 days. A correlation index of −0.97 was found between femur growth and decrease of bone tracer activity count between week 6 and 24.

**Conclusion:**

This methodological study demonstrates the potential of repeated bone planar scintigraphy in growing mice, with subcutaneous route for tracer administration, for quantitative assessment of bone remodeling.

## Introduction

Bone is a complex tissue, which is constantly renewed throughout the entire life. Bone remodeling is regulated by osteoblasts for bone formation and osteoclasts for bone resorption, with osteocytes being involved in bone balance regulation (Feng & McDonald, 2011). Long bones grow by endochondral ossification, which involves the formation of a cartilage, replaced by bone at a later stage, involving or preceded by osteoblast recruitment (Kronenberg, 2003; Rosello-Diez & Joyner, 2015).

To assess bone turnover, biochemical markers of bone resorption and formation in blood and urine may provide a global view (Shetty et al., 2016; Wheater et al., 2013). Histomorphometry, however, remains the gold standard to estimate local cortical and trabecular bone structure, bone mineralization, as well as osteoblast and osteoclast activity. The use of fluorochrome labeling allows for the calculation of bone remodeling parameters such as mineral apposition rates, mineralizing surface and bone formation rate (Recker et al., 2011). The major drawback of histomorphometry is the invasiveness of the biopsy. In clinical routine, repeated biopsies can’t easily be performed. Moreover, the biopsy site is not always representative of the bone structure of whole body.

In contrast, imaging technologies provide a non-invasive assessment of the bone tissue. Two different types of bone imaging are frequently used in clinical care: anatomical imaging with classically X-ray based modalities, high-resolution peripheral computed tomography, and functional imaging with bone scintigraphy (Ventura et al., 2014). Bone densitometry using dual-energy X-ray absorptiometry is commonly used for monitoring patients with osteoporosis (Blake & Fogelman, 2007; Blake, Frost & Fogelman, 2009a; Blake, Moore & Fogelman, 2009b). Bone planar scintigraphy offers a highly sensitive measurement of bone activity in pathologies involving the bone and allows for detection of many disease states, which may escape standard X-ray diagnosis (Bevan et al., 1980). Phosphonate tracers (hydroxymethylene diphosphonate, hydroxyethylene diphosphonate, 2,3-dicarboxypropane-1,1 diphosphonate) are commonly used for bone scintigraphy; their uptake depends on blood flow and on the rate of new bone formation, since phosphonates are mostly adsorbed to the osseous mineral phase (Brenner et al., 2012; Zhong et al., 2015). Whole-body planar scintigraphy provides a semi-quantitative assessment of bone uptake abnormalities in the entire bone body.

Mice are widely used in experimental studies, based on their gene modifiability, and providing insights in disease states such as acute and chronic renal failure, respective diagnostic and therapeutic interventions. To adequately assess bone health, quantitative measurements of bone turnover are essential. Histomorphometric studies which provide detailed information, can only be done *ex vivo*, but preclude repeated assessments and thus longitudinal follow-up. Currently, the ultimate tool for bone histomorphometry is high-resolution X-ray microcomputed tomography (µCT), which allows direct measurement of three-dimensional bone microarchitecture with a high spatial resolution of few micrometers *ex vivo*, and can also be used *in vivo*, with a lesser spatial resolution (Bouxsein et al., 2010; Feldkamp et al., 1989). It, however, does not give insight into bone turnover, or in complex experimental conditions (Schulte et al., 2011).

Surprisingly few studies have used planar scintigraphy to explore the bone in mice (Zhao et al., 2014), even though it is widely used in the clinical setting and provides information on bone turnover. In this article we present a methodology; we designed a quantitative evaluation of bone uptake of phosphonate tracer on knee’s regions of interest (ROI)—at the epiphyseal plate regions—drawn on bone planar scintigraphic images, as a measure of osteoblast activity. An index was calculated from the counts in knee’s ROI (normalized by pixels and seconds), corrected for activity administered, decay between administration and imaging, and individual animal weights. Since injection of radiotracer into veins is challenging in mice, and repetitive vein injections are hardly feasible we chose subcutaneous (SC) delivery for the tracer. To validate this quantitative approach, we concomitantly measured bone growth in healthy mice by µ-CT, as this natural process has widely been documented both in terms of function (osteoblast activities) and anatomy (bone length), with the latter being assessed by µ-CT.

## Methods

### Animal management

Eight C57Bl/6 mice (five males, three females from animal Facility Heidelberg) were longitudinally followed for 21 weeks (from age of 6 weeks just after weaning, to 27 weeks). Animals were housed in standard conditions: two individually ventilated cages, one for the male and the other for the female mice (SealSafe 1291H, Tecniplast, Italy) with environmental enrichment, 12 h light/dark cycle, controlled temperature and having *ad libitum* access to food and water.

All experiments were conducted according to EU regulations concerning small animal experimentation. The project was approved by local authorities (Regierungspräsidium Karlsruhe, Germany; Nr.35-9185.81 G-12/12). Euthanasia by cervical dislocation was performed 4 weeks after the end of the protocol and had been planned in case of bad tolerance but was not required.

### Imaging procedures

**Figure 1:**
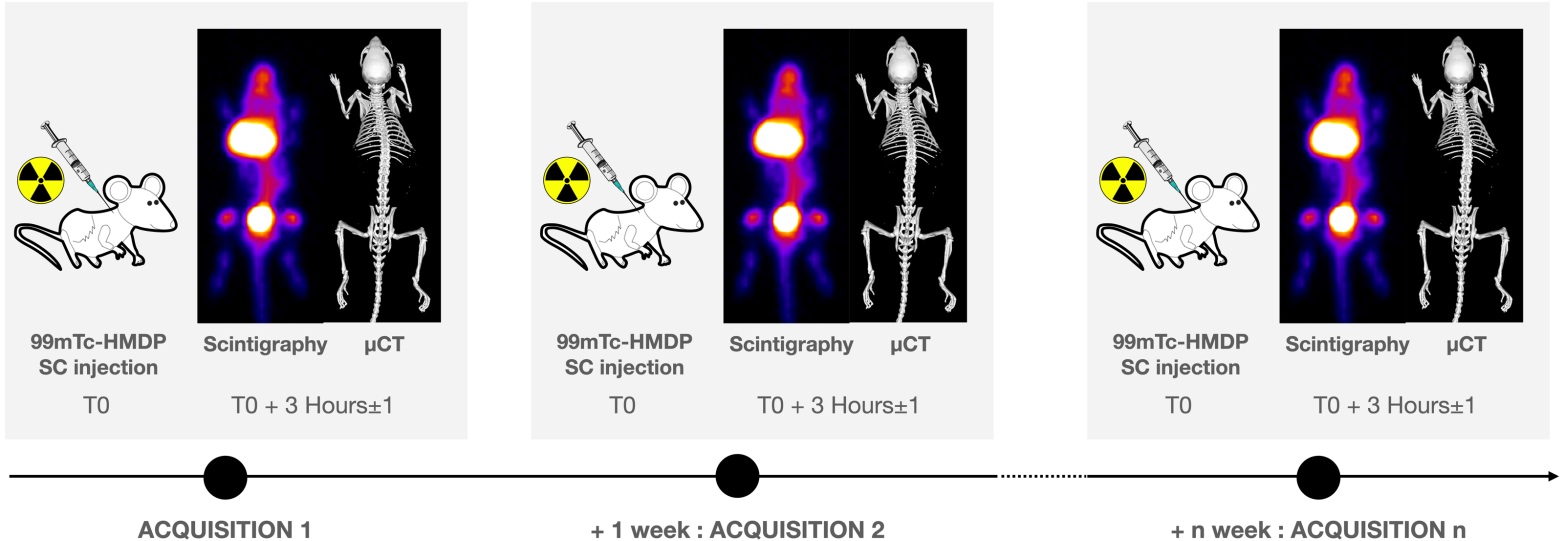
Experimental scheme.

**Figure 2:**
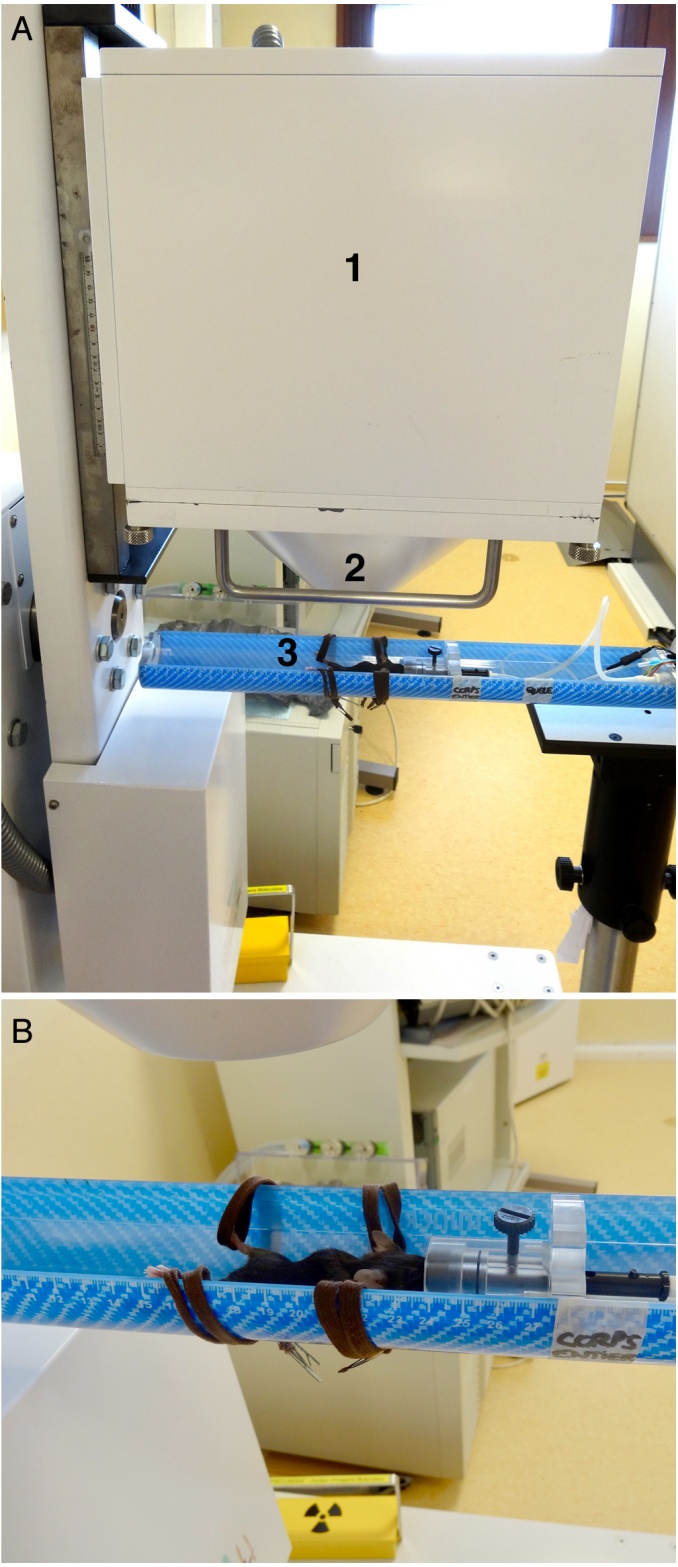
Experimental setup. (A) Side view, showing (1) the head of the gamma-camera, (2) the collimator and (3) the animal inside the bed. (B) Close view of the bed showing the laces used to move away the hind legs, and especially the knees, from the body.

**Figure 3:**
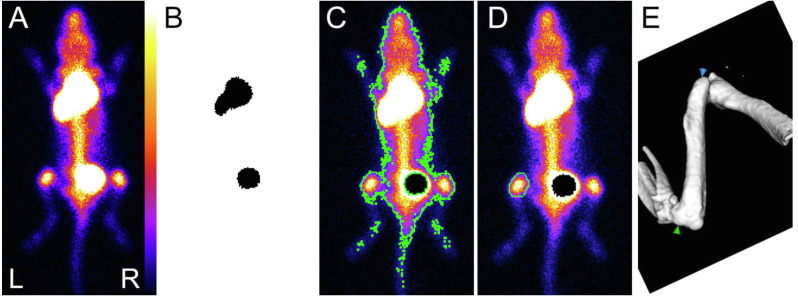
Sequential view of data processing. (A) Typical dorsal view, saturated pixels, allowing visualization from top to bottom the injection site, the bladder and the knees. (B) Automatically applied threshold using maximum entropy method: the injection site (top) and the bladder (bottom) were detected, and masks are used to define corresponding ROI. They are used to get the number of counts inside the injection site and to withdraw the bladder for the next step. (C) Automatically applied threshold using Huang’s fuzzy thresholding method to create the whole body ROI (surrounded by a green line). (D) Freehand ROI drawn on the left knee (surrounded by a green line). (E) Isosurface representation of the femur (selected from a typical whole body µCT acquisition): femoral length was measured between the higher point of greater trochanter (green arrow) and the maximal concavity of lateral epicondyle (blue arrow).

The imaging protocol included weekly acquisitions in the mice between the ages of 6 to 19 weeks, followed by three acquisitions in each mouse between ages of 19 to 27 weeks (Fig. 1). Each acquisition was performed at the same time of the day in order to reduce the potential influence of the nycthemeral cycle influence. For planar scintigraphy, a dedicated Anger type gamma camera (Gaede Medizinsysteme GmbH, Freiburg, Germany) was used with a pinhole collimator (hole diameter: two mm, focal length: 12 cm) and a NaI(Tl) scintillation crystal (6.5 mm in thickness). Acquired field of view (FOV) and matrix were 170 × 170 mm^2^ and in 256 × 256 respectively. The middle of the bed was always placed at a distance of 5.7 cm from the hole of the collimator: in these conditions, the projection of an adult mouse body encompasses the FOV (Fig. 2A). 120 ± 60 MBq of ^99m^Tc-hydroxymethylene diphosphonate (HMDP) (CIS bio international, Gif-sur-Yvette, France), prepared following the manufacturer’s recommendations and good practices, were administered under gaseous anesthesia (isoflurane 5% for induction followed by isoflurane 1.5 to 2% pushed by air) through neck SC injection (0.26 ± 0.22 ml), after weighing the animal. Volumes higher than 0.3 ml were delivered on two sites. These values are in the range considered as good practice for SC drug administration. ^99m^Tc-HMDP injections were performed before each acquisition. Since the half-life of the tracer is 6 h, no residual activity was detected at the beginning of the following acquisition. Activities of the tracer in the syringe before and after administration were measured (dose calibrator, CRC-25; Capintec Inc., Florham Park, USA) and the time of measurement recorded. After tracer administration, animals were allowed to wake up and were put back in a cage until image acquisition. After a delay of three (3 ± 1) hours, pinhole whole-body acquisitions (15 min) with a window of 140 keV ± 10%, were performed (Fig. 3A), keeping the individual under gaseous anesthesia (as before) in a warmed dedicated imaging cell (Minerve, Esternay, France) to enable homeostasis conditions (Fig. 2B). Quality control of the gamma camera was regularly done to assess a variation in response over time. For this purpose, we used a small plastic square box, filled with a constant volume of 2 ml of a ^99m^Tc solution with an activity comparable with that used in the experiment. The same acquisition conditions were applied with the box placed at the same position as the animal, inside the same bed (after having controlled the level).

µCT (eXplore 120 Vision; GE, Waukesha, WI, USA) was performed immediately after each planar scintigraphy, using the same setup for the animals. The imaging cell was moved from the gamma-camera to the µCT device within a few seconds. Reconstructed voxel size was 100 × 100 × 100 µm^3^. Due to technical difficulties the 15th and 16th µCT acquisition in the mice could only partially be accomplished.

### Post-processing analysis

This was done using ImageJ (Schneider, Rasband & Eliceiri, 2012). Freehand ROI were drawn on the right knee (Fig. 3D), and automatically reproduced in the mirror on the left knee. The ROI includes the growth plate area. The manual way of drawing ROI renders their size and position subject to intra- and inter-reader variability. The processing was performed independently by three different skilled investigators, and each investigator performed each procedure three times. The three investigators defined slightly different ROIs, but the resulting decay curves were similar. Counts (normalized by seconds and pixels) were corrected for activity administered, decay between administration and median imaging time, and individual weights, which lead to the index used to compare tracer bone uptake, expressed in unit of counts s^−1^ pixel^−1^ MBq^−1^ g^−1^. Attenuation was neglected, due to the thickness of knees. The mean value for the two knees was used, keeping only measurements with ratio between knee’s values between 0.9 and 1.1 (Fig. 3). An exponential decay was fitted to all processed data, *e.g.*, with the data from the 3 investigators (pro Fit 7, Quantum Soft, Switzerland) using the following equation:

(1) \begin{eqnarray*}x=A{e}^{(- \frac{t}{{T}_{A}} )}+C\end{eqnarray*}

where x refers to the index, A is a constant in unit of counts s^−1^ pixel^−1^ MBq^−1^ g^−1^, t time of each acquisition, T_A_ the time constant of decay of osteoblast activity and C the asymptote or residual bone activity in adult mice (in counts s^−1^ pixel^−1^ MBq^−1^ g^−1^). To check the robustness of the SC route, a ratio between ROI encompassing the whole body and ROI around the injection site was calculated. Both ROI were automatically delineated using automatic threshold methods included in ImageJ (Figs. 3B and 3C). Upon detection, the bladder content was withdrawn from the whole body ROI. Post processing of µCT data was done using Microview (version 2.5, Parallax Innovations, Ilderton, Canada). An isosurface rendering view of bones was generated with the marching cube algorithm, on which the femoral length was measured (Fig. 3E). A Gompertz law was fitted to the acquired data (pro Fit 7, Quantum Soft, Switzerland) using the following equation:

(2) \begin{eqnarray*}y=B{e}^{-be(- \frac{t}{{T}_{G}} )}\end{eqnarray*}

where y refers to the femur length, B is the asymptote or size of adult femur, b is representative of the time of onset of the phase of rapid skeletal growth as well as the time when growth ceases, t time of each acquisition, T_G_ the time constant of decay of growth (Rolian, 2008).

### Statistics

All results were expressed as means ± SD, or SEM when justified (*e.g.*, several calculations of same measurements) using Excel (Microsoft, Redmond, USA). Correlations were all calculated using Pearson correlation coefficient.

## Results

In order to validate the quantitative scintigraphy method in mice the following measurements were obtained. To assess the reproducibility, quality control of the gamma camera was performed twice per month over one year (including the time of this experiment). The coefficient of variation of the calculated index equaled 8% without a continuous increase or decrease of the index throughout the year. The tracer labelling was compliant with applicable standards.

The scintigraphy procedure in average required about 15 min for animal preparation, injection, and recovery tasks, and 15 min for data acquisition. A total of 93 scintigraphy studies and 85 µCT were accomplished. Three mice died, two (one male, one female) at the age of 12 and one female at the age of 14 weeks due to anesthesia related complications. The results are summarized in Table 1.

**Table 1:** Morphometric measurements  (weight and femur length measured by µCT) and scintigraphic index by gender and acquisition.

** **	** **	**Mean Body Weight ± SD (g)**			**Mean Femur length ± SD (mm) Number (N**)			Mean Scintigraphic index ± SD (counts s^−1^ pixel^−1^ MBq ^−1^ g^−1^× 10^**6**^ ) Number (N)		
**Acq.**	**Days (Weeks)**	**Female**	**Male**	**All**	**Female**	**Male**	**All**	**Female**	**Male**	**All**
**1**	43 (6.1)	13.3 ± 0.6	14.6 ± 2.9	**14.1 ± 2.4**	11.82 ± 0.47(3)	12.41 ± 0.87(5)	**12.19 ± 0.77**	86.4 ± 8.1(3)	91.8 ± 13.7(5)	**87.4 ± 2.6**
**2**	52 (7.4)	16.7 ± 1.5	18.5 ± 2.1	**17.4 ± 1.82**	13.15 ± 0.35(3)	13.44 ± 1.06(2)	**13.47 ± 0.53**	67.7 ± 14.3(3)	63.7 ± 10.0(2)	**66.0 ± 5.4**
**3**	58 (8.3)		22.0 ± 1.0	**22.0 ± 1.0**		13.95 ± 0.57(2)	**13.95 ± 0.57**		34.0 ± 3.7(3)	**34.0 ± 3.7**
**4**	67 (9.6)	19.0 ± 0	22.0 ± 0.7	**20.88 ± 1.6**	14.08 ± 0.16(3)	14.58 ± 0.37(5)	**14.39 ± 0.39**	49.3 ± 2.4(3)	27.3 ± 1.3(2)	**39.9 ± 2.6**
**5**	73 (10.4)	18.3 ± 0.6	22.2 ± 1.8	**20.75 ± 2.4**	14.32 ± 0.14(3)	14.77 ± 0.35(5)	**14.60 ± 0.36**	35.8 ± 3.8(3)	26.1 ± 7.5(5)	**30.5 ± 3.3**
**6**	80 (11.4)	19.0 ± 0	23.4 ± 1.1	**21.75 ± 2.4**	14.52 ± 0.13(3)	14.96 ± 0.37(5)	**14.90 ± 0.37**	43.6 ± 1.5(3)	27.6 ± 4.5(5)	**33.8 ± .0.3**
**7**	87 (12.4)	18.7 ± 0.6	23.8 ± 1.8	**21.88 ± 3.0**	14.70 ± 0.10(3)	15.16 ± 0.37(5)	**14.99 ± 0.37**	34.0 ± 1.7(3)	21.9 ± 4.1(5)	**26.8 ± 2.2**
**8**	94 (13.4)	19.5 ± 0.7	23.8 ± 2.2	**22.33+2.8**	14.83 ± 0.13(2)	15.20 ± 0.33(4)	**15.08 ± 0.33**	24.9 ± 0.6(2)	19.1 ± 6.2(4)	**20.7 ± 1.9**
**9**	101 (14.4)	20.0 ± 0	24.8 ± 1.5	**23.17 ± 2.7**	14.90 ± 0.04(2)	15.40 ± 0.39(4)	**15.23 ± 0.40**	33.4 ± 0.7(2)	16.8 ± 2.6(4)	**21.5 ± 3.5**
**10**	115 (16.4)	21.0	24.8 ± 3.3	**24 ± 3.3**	15.39(1)	15.50 ± 0.36(4)	**15.48 ± 0.31**	20.5(2)	16.7 ± 3.0(4)	**17.6 ± 2.0**
**11**	123 (17.6)	21.0	25.8 ± 1.3	**24.8 ± 2.4**	15.46(1)	15.64 ± 0.35(4)	**15.60 ± 0.32**	11.4(1)	8.5 ± 0.9(4)	**9.2 ± 0.9**
**12**	130 (18.6)	21.0	26.5 ± 0.6	**25.4 ± 2.5**	15.50(1)	15.57 ± 0.29(4)	**15.55 ± 0.26**	9.4(1)	7.4 ± 1.3(4)	**7.5 ± 0.9**
**13**	136 (19.4)	21.0	26.8 ± 2.2	**25.6 ± 3.2**	15.61(1)	15.58 ± 0.28(4)	**15.59 ± 0.25**	17.9(1)	16.0 ± 3.8(4)	**16.2 ± 0.7**
**14**	157 (22.4)	22.0	28.0 ± 1.8	**26.8 ± 3.1**	15.69(1)	15.83 ± 0.24(4)	**15.80 ± 0.22**	12.9(1)	9.8 ± 7.8(4)	**11.5 ± 0.9**
**15**	171 (24.4)	22.0	27.2 ± 1.5	**26.2 ± 2.7**		15.85(1)	**15.85**	23.5(1)	13.8 ± 2.3(4)	**16.4 ± .1.5**
**16**	192 (27.4)	23.0	27.2 ± 1.0	**26.4 ± 2.1**			** **	17.6(1)	11.2 ± 0.8(4)	**15.0 ± 3.3**

**Notes.**

Acq Acquisition

**Figure 4:**
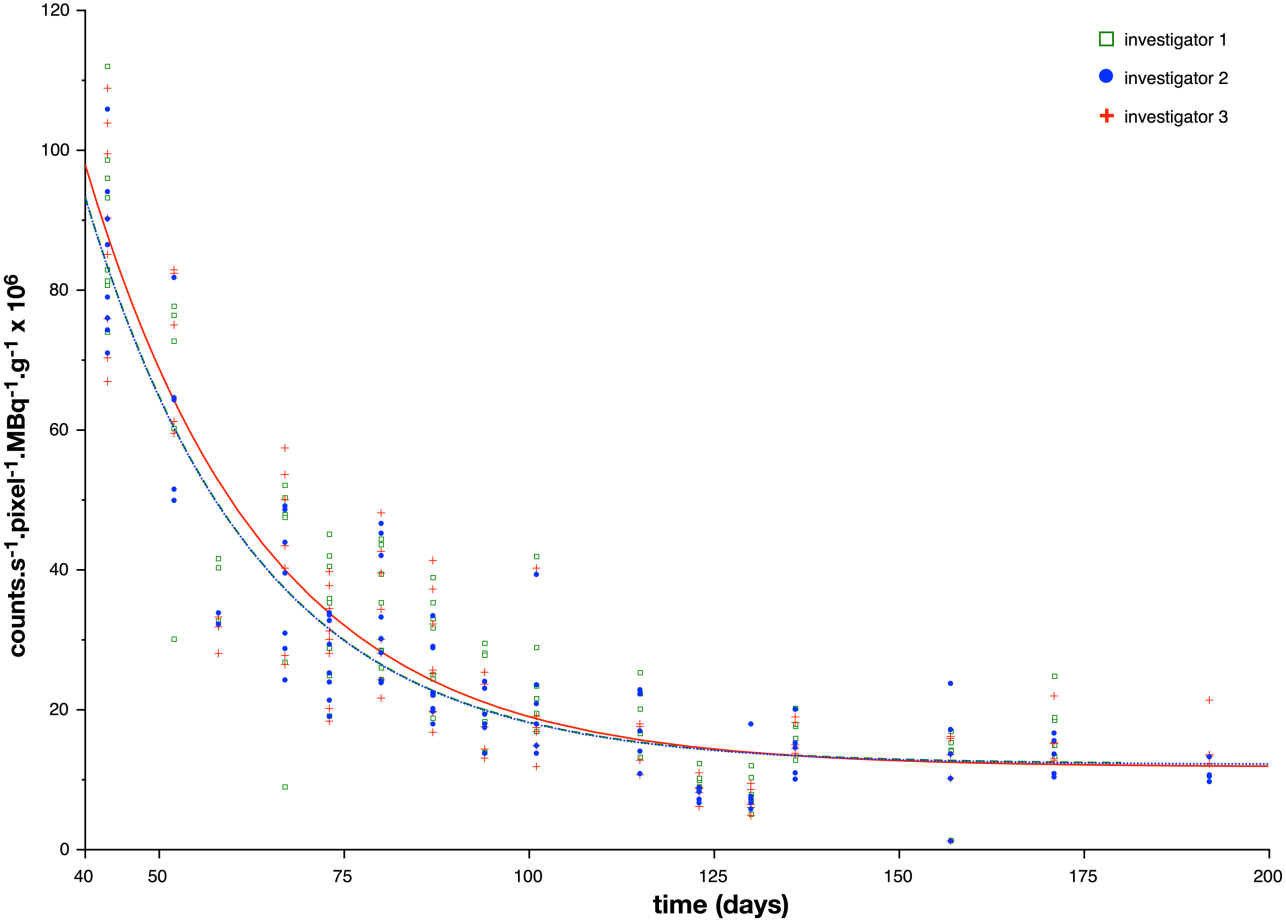
Index reflecting bone planar scintigraphic activity as a function of the age of the animals. A color dot (one color per investigator) represents one processed acquisition. The three lines represent the exponential formula (1) used to fit scintigraphic data. A table (Table 2) is given with the formula for each investigator.

**Table 2:** Table associated with Fig. 4.

Results in mean ± standard deviation	Investigator 1	Investigator 2	Investigator 3
A in counts s^−1^ pixel^−1^ MBq^−1^ g^−1^ × 10^6^	468.4 ± 99.2	462.1 ± 82.6	451.2 ± 84.7
T_A_ in days	23.2 ± 2.6	23.0 ± 2.1	24.2 ± 2.5
C in counts s^−1^ pixel^−1^ MBq^−1^ g^−1^ × 10^6^	14.4 ± 2.1	12.3 ± 1.6	11.9 ± 2.1

The tracer activity was measured in the left and right knees. The mean corrected counts in the knee region decreased from 87.4 ± 2.6 ×10^−6^ counts s^−1^ pixel^−1^ MBq^−1^ g^−1^ at week 6 to 15.0 ± 3.36 × 10^−6^ counts s^−1^ pixel^−1^ MBq^−1^ g^−1^ at week 27 ( Table 1 and Fig. 4). The correlation indexes between the 3 investigators for the mean corrected counts were 0.99. The mean parameters of the fitted exponential decay were: *A* = 460.6 × 10^−6 ^ counts s^−1^ pixel^−1^ MBq^−1^ g^−1^ and the time constant T_A_ of the exponential decay was found equal to 23.5 days. For calculation, C, asymptote or residual bone activity in adult mice, was chosen equal to the mean of the 16th measurements (15.0 × 10^−6 ^ counts s^−1^ pixel^−1^ MBq^−1^ g^−1^) (Table 1 and Fig. 4). The mean ratio calculated between the whole body ROI and the SC site of injection, for all acquisitions was equal to 6.84 ± 1.84.

**Figure 5:**
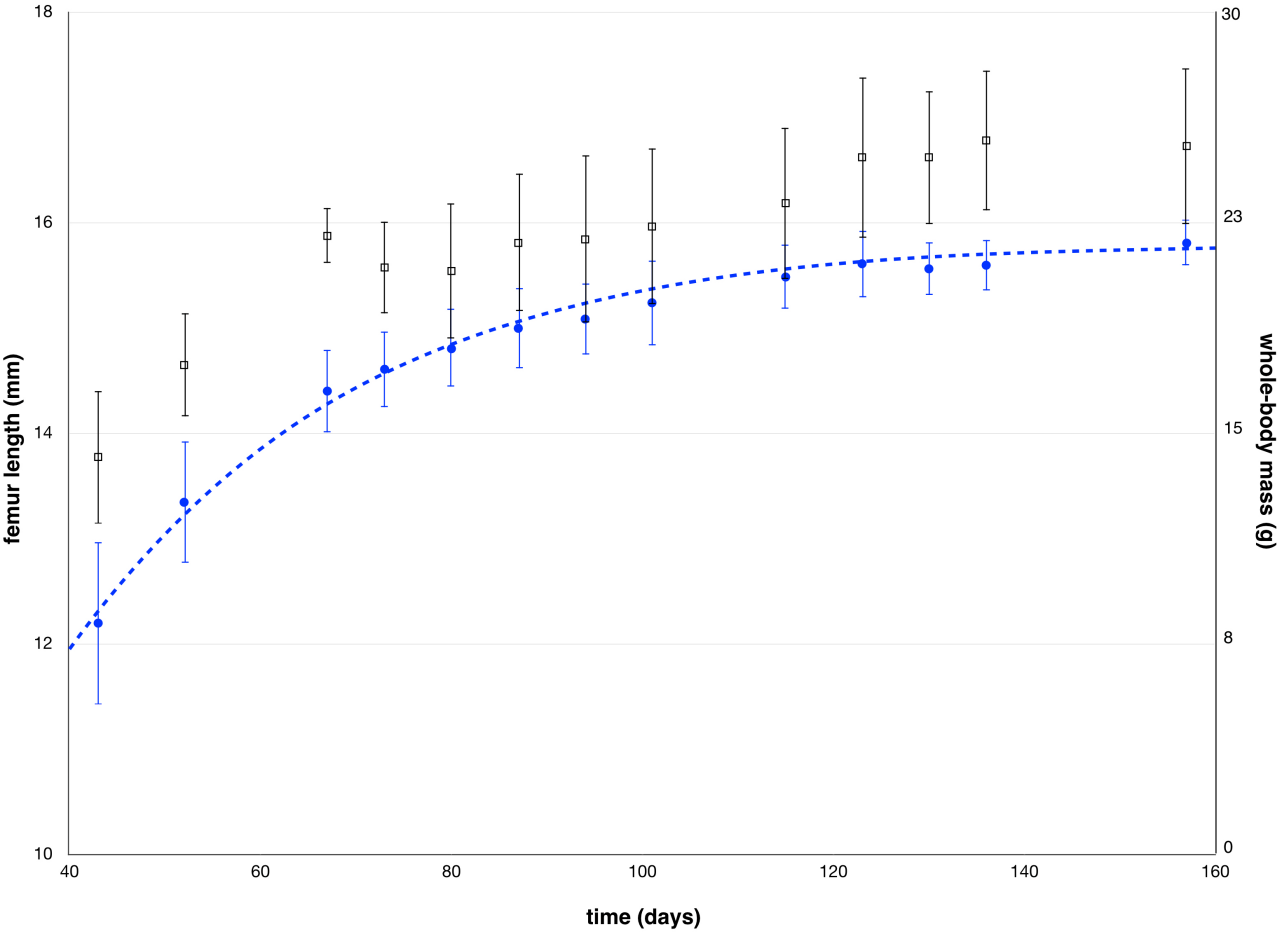
Mean values of femur length measured by µCT and mean values of whole-body mass as a function of the age of the animals, along with standard deviations. The blue dotted line represents the Gompertz formula (2) used to fit femur length.

To further validate our model, we measured femur length after each scintigraphy study by µCT. Mean femur length increased from 12.2 ± 0.8 mm at week 6 to 15.8 ± 0.2 mm at week 22 (Fig. 5). The parameters of the fitted Gompertz law were: *b* = 1.25 and T_G_ the time constant of the decay of growth equaled 26.7 days. For calculation, B was chosen equal to the mean of the 14th measurements (15.8 mm) (Table 1).

**Figure 6:**
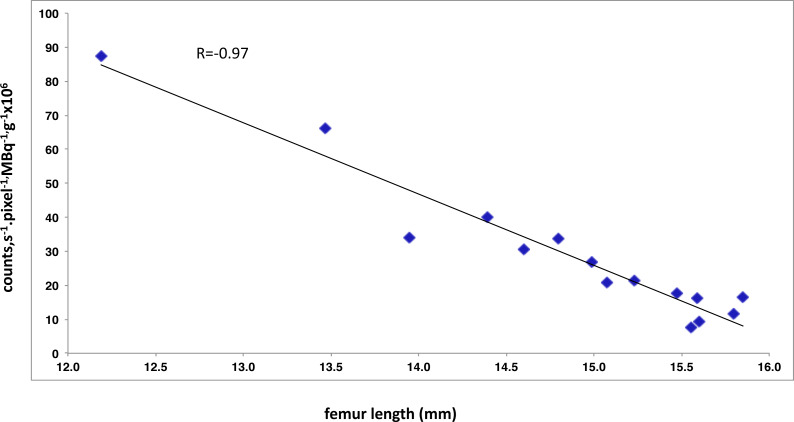
Negative correlation between the index reflecting bone planar scintigraphic and the mean femur length measured by µCT. Pearson correlation coefficient, *r*, is given is the upper part of the scatter plot.

A correlation index of −0.97 was found between femur growth and decreased of bone tracer activity count between week 6 and 24 (Fig. 6).

## Discussion

Despite widespread use of bone planar scintigraphy in patients, its application in mice remains anecdotal (Khairnar et al., 2015; Tin Ong et al., 2008), even though data on bone turnover can be obtained, which otherwise require bone biopsies. We now demonstrate that frequent quantitative planar scintigraphy with subcutaneous tracer injection is feasible in mice and allows for frequent longitudinal assessment of bone turnover during the growing period. We demonstrate a decrease in phosphate tracer counts that matched with an exponential decay, and in parallel a respective decline in longitudinal growth rate that could be matched with a Gompertz law. A strong negative correlation was found between the two variables, indicating that our bone planar scintigraphy index reflects bone longitudinal remodeling dynamics.

The scintigraphy procedure required 15 min for animal preparation, injection, and recovery tasks only, and 15 min for scintigraphic data acquisition. The subcutaneous injections were well tolerated and allowed for adequate tracer studies. Despite the high number of imaging procedures only three mice died, all due to anesthesia related complications, none due to imaging related complications.

In the clinical setting diphosphonate tracer are widely used as phosphate analogs, which complex with the crystalline hydroxyapatite in the mineral phase of bone. They localize to bone in proportion to osteoblastic activity, but also to some extent in relation to blood perfusion and thus tracer delivery (Adams & Banks, 2020). Bone metastasis but also different osteoblastic and hyperostotic lesions can be detected but often require additional imaging for diagnosis (Ju & Paycha, 2020). We now demonstrate the feasibility of frequent diphosphonate nuclear scan studies in C57Bl/6 mice, a mouse strain widely used for *in vivo* experimental studies, and the validity by comparing the results to femur growth using concomitant µCT studies. Our findings are also in line with previously reported longitudinal growth and osteoblast activity pattern in C57Bl/6 mice: Brodt et al. described rapid femoral growth during week 4 and 8 of life, followed by slower growth between weeks 8 and 12 (Brodt, Ellis & Silva, 1999). Chan et al. reported an increase in femur length until a plateau at day 60 (Chan et al., 2012). Ferguson et al. (2003) demonstrated rapid femur and growths in male C57BL/6 mice, from 4 to 22 weeks of age, after which longitudinal bone growth abated. Bone formation rate and mineral apposition rate rapidly decreased with aging with an important slope until 12 weeks of age (Ferguson et al., 2003). Other mice strains exhibited a decline in bone formation assessed by fluorochromes from week 6 to week 26 of life and a decrease in mineral apposition and bone formation rate in the femur between 28 and 60 days of life (Gardinier et al., 2018; Sheng et al., 1999; Tsuboyama et al., 1989).

Our results are consistent with all these previous studies. The femur grew rapidly from week 6 to week 10, followed by growth deceleration until week 19, and a small but steady growth rate thereafter. In line with this, scintigraphic counts, a surrogate for femur osteoblast activity, declined with a declining growth velocity. Our findings also fit to histomorphometry labeling findings in mice (Sheng et al., 1999), a direct comparison with scintigraphy studies, to our knowledge has not yet been obtained. In contrast to such labeling measurements, our method has the major advantage of being repeatedly performed *in vivo*. Our method complies with two of the three ethic “Rs” tenet for experimental animal use, replacement, reduction, and refinement (Russell & Burch, 1959), as fewer animals are required and follow-up of the same animal increases statistical power of a study. It provides an alternative to the sacrifice of animal for dynamic bone study in future experiments.

Our findings should also be transferable to rats, which grow from birth to 200 days of life (Sontag, 1986a; Sontag, 1986b; Sontag, 1992; Sontag, 1994) and to some extent to humans (Glorieux et al., 2000; Rauch, Travers & Glorieux, 2006), where dynamic histomorphometry data and planar scintigraphy data have been obtained in children, adolescents and young adults. Brunot et al. described an age dependent lower bone tracer uptake in 82 boys and 36 girls age 1 to 21.5 years using quantitative ^99m^Tc-HMDP planar scintigraphy (Brunot et al., 1991). Other quantitative radionuclide studies in growing children support these results (Celen et al., 1999; Yamane et al., 2018).

Precise quantitative planar scintigraphy in humans is based on repeated blood sampling or repeated acquisition over time (Blake, Moore & Fogelman, 2009b; Brenner et al., 1997; D’Addabbo et al., 1992). This approach can hardly be transferred to mice models, as blood sampling is limited in quantity and repeated acquisitions require extended anesthesia. Our approach is less sophisticated but repeatedly feasible in live mice and still provides meaningful data.

Very few studies of bone growth tried to extract global parameters based on a mathematical model, fitted to acquired data. And it is even more challenging to get the respective numerical values. In Rolian (2008), for the femur, the corresponding Gompertz law parameters were b = 1.44 and T_G_ = 14.0 days. Beyond the order of magnitude, comparison of values with our findings is hampered by the fact that this study was done *ex vivo*, started immediately after birth and was discontinued at 85 days of life and included females only. To our knowledge there is no experimental study providing a mathematical model to match osteoblastic activity over time in mice. The time constants are in the same order, with T_G_ being longer than T_A_.

Repeated intravenous tracer injections in the mice tail are difficult to perform and result in reduced reproducibility as previously described with quantitative PET (Lasnon et al., 2015; Vines et al., 2011). The small caliber of the rodent vein can lead to extravasation of the tracer and incorrect interpretation of imaging. Correction factors for extravasation have been developed but do not sufficiently solve this issue (Lasnon et al., 2015). Intraperitoneal administration of radiotracer may be used instead, but again injection quality is poor in 10 to 20% of cases and is associated with risk of tissues injuries. Moreover pharmacokinetics obtained with IP administration are more akin to those after oral administration as compared to the intravenous route (Fueger et al., 2006; Lukas, Brindle & Greengard, 1971; Schiffer, Mirrione & Dewey, 2007; Vines et al., 2011). Depending on their pharmacokinetics, some tracers cannot be administered intraperitoneally. Retroorbital administration is not approved by all animal committees and cannot be repeated frequently (Vines et al., 2011). We therefore chose the SC route, where the tracer is injected into the extracellular space and will be transported to blood or lymph for absorption (Richter & Jacobsen, 2014). Comparing to the intravenous route, the maximal plasma concentration may be reached later and may be lower as found for contrast-enhanced magnetic resonance imaging (Dillenseger et al., 2019), but pharmacokinetics and pharmacodynamics of radionuclide tracers injected subcutaneously have not been studied systematically. Tolerance of the SC injections was good, no signs of inflammation at the injection site were observed. This is in contrast to previous findings (Lim et al., 2010), but may be explained by different preparation of ^99m^Tc-HMDP, which in our case has a pH of about 6. The use of SC route was accurate in our study given the shape of the scintigraphic curve.

Our study has some limitations. We used female and male mice in a small group of animals, which precludes firm conclusions on gender specific differences. However, the fact that imaging was repeatedly performed in the same animals should minimize this bias and the purpose of the study was to establish frequent planar scintigraphy measurements and relate findings to growth rate and not to detect gender specific bone related differences. Similar growth pattern was found in both genders in C57Bl/6 mice with comparable final femur length (Glatt et al., 2007). Both genders tolerated the procedure well. Of note, to reproduce our findings using another planar scintigraphy device would require an independent and precise calibration protocol again and longitudinal studies should be accomplished using always the same device. The parameters extracted from the mathematical model could, of course still be compared, regardless which gamma camera is used. Numerical results may depend on the genetic background of the strain, specific reference values should therefore be considered. In case, frequent bone scintigraphy (and µCT studies) are applied together with molecular bone tissue studies, an impact of the repeated radiation exposure on molecular expression profiles cannot be excluded.

## Conclusions

We have developed a simple and fast quantitative planar scintigraphy method in mice, for repeated, longitudinal assessment of bone dynamics in growing mice. The subcutaneous route of tracer application provided adequate tracer kinetics, and the findings were in line with concomitant µCT data of femur length growth. Our method may be applied to establish respective normal values of bone turnover in healthy mice and to compare these to respective findings in mice with various types of bone disease.

## Supplemental Information

10.7717/peerj.12355/supp-1 Supplemental Information 1 Raw data for figures

10.7717/peerj.12355/supp-2 Supplemental Information 2 Author Checklist

## Additional Information and Declarations

### Data Availability

The following information was supplied regarding data availability: The raw measurements for figures are available in the Supplementary File.
